# A Case of Duodenal “Spot” Diagnosis

**DOI:** 10.7759/cureus.25500

**Published:** 2022-05-30

**Authors:** Hira I Cheema, Saikiran Raghavapuram, Iman Boston, Thomas Augustine, Benjamin Tharian

**Affiliations:** 1 Internal Medicine, Baptist Health Medical Center, Little Rock, USA; 2 Gastroenterology, University of Arkansas for Medical Sciences, Little Rock, USA

**Keywords:** pigmentation disorders, endoscopy, duodenum, pseudomelanosis, iv iron

## Abstract

Pseudomelanosis duodeni is a rare finding usually described as a black/brown speckled or tattooed appearance of the intestinal mucosa. Although an incidental finding, it has been associated with different medications and chronic medical conditions such as diabetes mellitus and chronic renal failure. We describe an elderly male who presented with epigastric pain and melena. Endoscopy showed pseudomelanosis duodeni related to intravenous (IV) iron transfusion. To our knowledge, this is the first report of pseudomelanosis duodeni related to IV iron use. In spite of its benign nature, the diagnosis of pseudomelanosis duodeni is essential to rule out other serious medical conditions that mimic its physical findings.

## Introduction

Pseudomelanosis duodeni has been reported with chronic medical conditions such as diabetes mellitus, hypertension, end-stage renal disease, post renal transplant, and with associated therapies in these conditions [1]. Oral iron therapy has been associated with pseudomelanosis but there are no reports of this condition with IV iron transfusion [2].

## Case presentation

A 75-year-old male with a history of iron deficiency anemia presented with ongoing melena for six months. He was receiving outpatient iron transfusions for his anemia. Hemoglobin levels were stable with transfusions. He did not have any other pertinent medical history and was not using any blood thinners. The patient denied any smoking, alcohol, or recreational drug abuse. Esophagogastroduodenoscopy (EGD) and colonoscopy as outpatient were unremarkable. Capsule endoscopy showed questionable arteriovenous malformation in the jejunum. The patient was referred for balloon-assisted enteroscopy (BAE). The patient’s symptoms had resolved one week prior to the procedure. BAE showed diffuse blue pigmentation in the duodenum and as such no arteriovenous malformations (AVMs) were seen to the extent small bowel was examined up to mid jejunum (Video 1). 

**Video 1:** Normal duodenum (left) as compared to pigmented appearance in pseudomelanosis duodeni (right)

**Figure 1:**
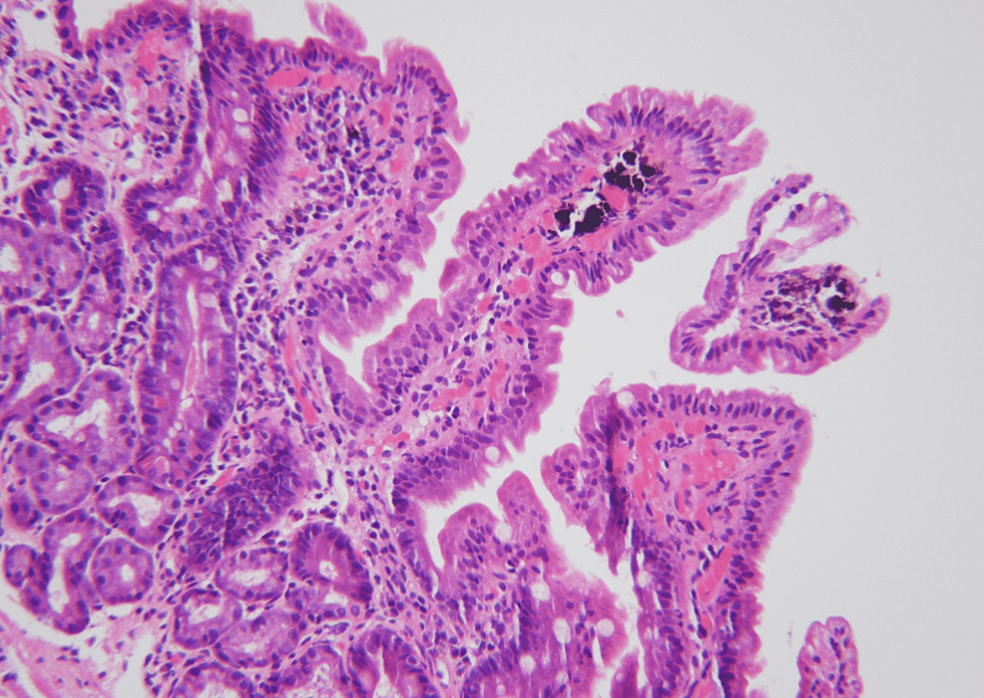
Pseudomelanosis duodeni - histological examination showed brown/black pigmentation in the tip of the villi.

The differential diagnosis for such a finding includes iron pill duodenitis, pseudomelanosis duodeni, eosinophilic enteritis, hemochromatosis, clofazimine-related pigmentation, or rarely malignant melanoma. All of these harbor a worse prognosis than pseudomelanosis duodeni and histopathology is required for accurate diagnosis. Our biopsy findings confirmed pseudomelanosis showing deposition of brown pigment within macrophages in the lamina propria of normal villi (Figure 1). 

## Discussion

Pseudomelanosis duodeni was first identified in 1976 and was described as a form of stored iron in duodenal villi [3]. On histological examination, black pigments are located in the lysosomes of macrophages in the lamina propria of the gastrointestinal tract [4]. It is associated with chronic medical conditions such as diabetes mellitus, chronic renal disease, essential hypertension, and in related therapies such as sulfur-containing diuretics or iron [5]. Several mechanisms have been proposed for its pathogenesis. These theories include mechanisms such as iron deposition as a result of intramucosal hemorrhage or impaired luminal iron transport after oral supplementation [6]. As proximal duodenum is the site of maximum iron absorption during normal digestion, it is not surprising to see abundant pigmentation in this part of small bowel. Some suggest that the pigment may be a combination of melanin-like substances, lipomelanin, hemosiderin, and lipofuscin [3,6]. Certain drugs such as propranolol, hydralazine, hydrochlorothiazide, and furosemide have also been related to pseudomelanosis duodeni in some reports [7,8]. However, intravenous iron has not been directly linked to the development of pseudomelanosis duodeni. 

Our patient was not on any oral therapies that have been linked to pseudomelanosis duodeni neither did he have any of the chronic comorbidities. It is inciting to see that iron supplementation even as an intravenous route could develop this condition.

## Conclusions

Pseudomelanosis duodeni is a rare benign condition that needs prompt recognition and histological diagnosis. It is important to rule out other differentials and pursue appropriate investigations. Our case sheds light on a risk factor that has not been reported before, i.e., intravenous iron supplementation.
